# Dynamic A-to-I RNA editing during acute neuroinflammation in sepsis-associated encephalopathy

**DOI:** 10.3389/fnins.2024.1435185

**Published:** 2024-08-02

**Authors:** Yu-Ning Li, Ya-Ping Liang, Jing-Qian Zhang, Na Li, Zhi-Yuan Wei, Yijian Rao, Jian-Huan Chen, Yun-Yun Jin

**Affiliations:** ^1^School of Biotechnology, Jiangnan University, Wuxi, China; ^2^Laboratory of Genomic and Precision Medicine, Wuxi School of Medicine, Jiangnan University, Wuxi, Jiangsu, China; ^3^Wuxi Maternal and Child Healthcare Hospital, Wuxi, Jiangsu, China

**Keywords:** RNA editing, sepsis-associated encephalopathy, acute neuroinflammation, cerebral vessels, cerebral endothelial cells, microglia

## Abstract

**Introduction:**

The activation of cerebral endothelial cells (CECs) has recently been reported to be the earliest acute neuroinflammation event in the CNS during sepsis-associated encephalopathy (SAE). Importantly, adenosine-to-inosine (A-to-I) RNA editing mediated by ADARs has been associated with SAE, yet its role in acute neuroinflammation in SAE remains unclear.

**Methods:**

Our current study systematically analyzed A-to-I RNA editing in cerebral vessels, cerebral endothelial cells (CECs), and microglia sampled during acute neuroinflammation after treatment in a lipopolysaccharide (LPS)-induced SAE mouse model.

**Results:**

Our results showed dynamic A-to-I RNA editing activity changes in cerebral vessels during acute neuroinflammation. Differential A-to-I RNA editing (DRE) associated with acute neuroinflammation were identified in these tissue or cells, especially missense editing events such as S367G in antizyme inhibitor 1 (*Azin1*) and editing events in lincRNAs such as maternally expressed gene 3 (*Meg3*), *AW112010*, and macrophage M2 polarization regulator (*Mm2pr*). Importantly, geranylgeranyl diphosphate synthase 1 (*Ggps1*) and another three genes were differentially edited across cerebral vessels, CECs, and microglia. Notably, Spearman correlation analysis also revealed dramatic time-dependent DRE during acute neuroinflammation, especially in GTP cyclohydrolase1 (*Gch1*) and non-coding RNA activated by DNA damage (*Norad*), both with the editing level positively correlated with both post-LPS treatment time and edited gene expression in cerebral vessels and CECs.

**Discussion:**

The findings in our current study demonstrate substantial A-to-I RNA editing changes during acute neuroinflammation in SAE, underlining its potential role in the disease.

## Introduction

Sepsis-related encephalopathy (SAE) is an acute progressive brain dysfunction caused by systemic inflammation without direct CNS infection (Kuperberg and Wadgaonkar, 2017). SAE is a common complication of sepsis, affecting up to 70% of patients, which can range from mild confusion to coma and is a significant predictor of mortality (Gofton and Young, 2012). Cerebrovascular dysfunction plays a vital role in the development of SAE. Sepsis triggers a cascade of inflammatory events that can damage the blood vessels in the brain, leading to impaired blood flow and oxygen delivery to brain tissue, and microglia activation predominates neuroinflammation (Xin et al., 2023). Animal studies on SAE have shown that the activation of cerebral endothelial cells (CECs) is the earliest event in the CNS during the onset of acute neuroinflammation, suggesting a substantial role for CECs in SAE pathogenesis.

Although the underlying mechanisms of SAE remain largely unclear, our previous study implicated a possible link between RNA editing and SAE (Zhang J.-Q. et al., 2022). The adenosine-to-inosine (A-to-I) editing mediated by the adenosine deaminase acting on RNA (ADAR) protein family (Gallo et al., 2017) in mammals plays an important function in neurodevelopment and neuropsychiatric diseases (Krestel and Meier, 2018; Gumpper et al., 2022), such as amyotrophic lateral sclerosis (ALS), developmental epileptic encephalopathy, and depression (Yang et al., 2021). Recent studies have implicated that *ADAR* could be involved in sepsis (Shangxun et al., 2020), and A-to-I RNA editing could possibly be linked to SAE, which could possibly be attributed to its importance in inflammation. *ADAR* expression is upregulated during acute inflammation (Yang et al., 2003). *ADAR* and its mediated A-to-I are involved in inflammation (Sun et al., 2021). In humans, ADAR deficiency in humans and mice could cause Aicardi–Goutières syndrome (AGS), which is a severe autoinflammatory disease (Rice et al., 2012; Nakahama et al., 2021). ADAR prevents autoinflammation by inhibiting the activator of apoptosis and necroptosis (de Reuver et al., 2022). Nevertheless, the role of RNA editing in SAE, especially during the onset of acute neuroinflammation, remains to be elucidated.

Herein, our current study conducted an epitranscriptomic analysis of A-to-I RNA editing and revealed dynamic RNA editing in cerebral vessels, CECs, and microglia during acute neuroinflammation in a mouse SAE model.

## Materials and methods

### RNA-Seq dataset retrieval

Raw RNA-Seq read data was downloaded from the NCBI Gene Expression Omnibus (GEO)^1^ database. The sequencing data of mouse cerebral vessels (GSE155516) used for RNA editing analysis contained a control group treated with PBS and treatment groups treated with 10 mg/kg lipopolysaccharide (LPS) for 15 min, 30 min, and 4 h (*N* = 3 for each group). The sequencing data of mouse CEC and microglia (GSE155517) contained a control group treated with PBS and treatment groups treated with LPS for 30 min, 1 h, and 2 h (*N* = 3 for each group) (Kodali et al., 2021).

### RNA-Seq data processing and gene expression quantification

The raw sequencing data obtained above were analyzed following a pipeline as previously reported (Tao et al., 2021). In brief, Reads were aligned and mapped to the mouse genome (UCSC mm10) using RNA STAR (version 2.7.0e) (Dobin et al., 2013). Samtools (version 1.17) was used to filter the reads (Li et al., 2009). Base quality score recalibration was then performed with the resulting BAM files using GATK (version 4.1.3) and following the best practices workflows recommended by the documentation (Walker et al., 2018). Alignment files generated by RNA STAR were analyzed using FeatureCounts to obtain gene expression counts (Liao et al., 2014), and normalized gene expression levels (TPM) were calculated with edgeR (version 3.7) (Robinson et al., 2010).

### Identification of high-confidence A-to-I RNA editing events

Single nucleotide variation (SNV) identification was performed using VarScan (version 2.4.4) (Koboldt et al., 2012) as previously reported (Zhang J.-Q. et al., 2022). Filtering criteria were set as base quality ≥25, sequencing depth ≥ 10, alternative allele depth ≥ 2, and frequency ≥ 1%, and false positive variants were filtered and removed using VarScan version 4.4 with default parameters. Further, high-confidence variants were retained, defined as those with editing levels ≥1% detected in at least two samples or annotated as known RNA editing variants in the REDIportal database (Mansi et al., 2021). SNVs were then annotated using the Ensembl Variant Effect Predictor (VEP)^2^ (McLaren et al., 2016).^3^

### Enrichment analysis of gene functions and pathways

To understand the potential biological effects of RNA editing, Enrichr^4^ was used to analyze the gene ontology (GO) and Kyoto encyclopedia of genes and genomes (KEGG) pathways enriched by edited genes and false discovery rate (FDR) < 0.05 was used as the cut-off (Kuleshov et al., 2016).

### Statistical analysis

Comparison of RNA editing levels or gene expression levels between samples was performed using the ANOVA test or generalized linear model method and the likelihood ratio test to calculate the empirical *p-*values. Tukey’s Honest Significant Difference (HSD) test was used for post-hoc analysis between groups, and Benjamini-Hochberg correction was used for multiple comparisons. For RNA editing events with empirical GLM *p* < 0.05, an additional *Fisher*’s exact test was used to calculate Fisher’s *P* for intergroup comparisons of the total counts of the reference and alternative alleles among the four time-point groups. The Benjamini-Hochberg method was used to FDR adjustment of empirical *p*-values for multiple comparisons of RNA editing. RNA editing events were only considered to be differentially edited if both its (1) empirical GLM *p* < 0.05 and (2) GLM FDR < 0.05 or Fisher’s exact test FDR < 0.05. Principal component analysis (PCA) analysis was performed using the function Prcomp in R (version 3.6.3) software, and data visualization was performed using the ggplot2 (version 2.2.1) package (Tang et al., 2020). The correlation coefficient *r* and *p*-values were calculated using Spearman correlation analysis.

## Results

### A-to-I RNA editing activity changes in cerebral vessels, CECs, and microglia during acute neuroinflammation in SAE

**Figure 1:**
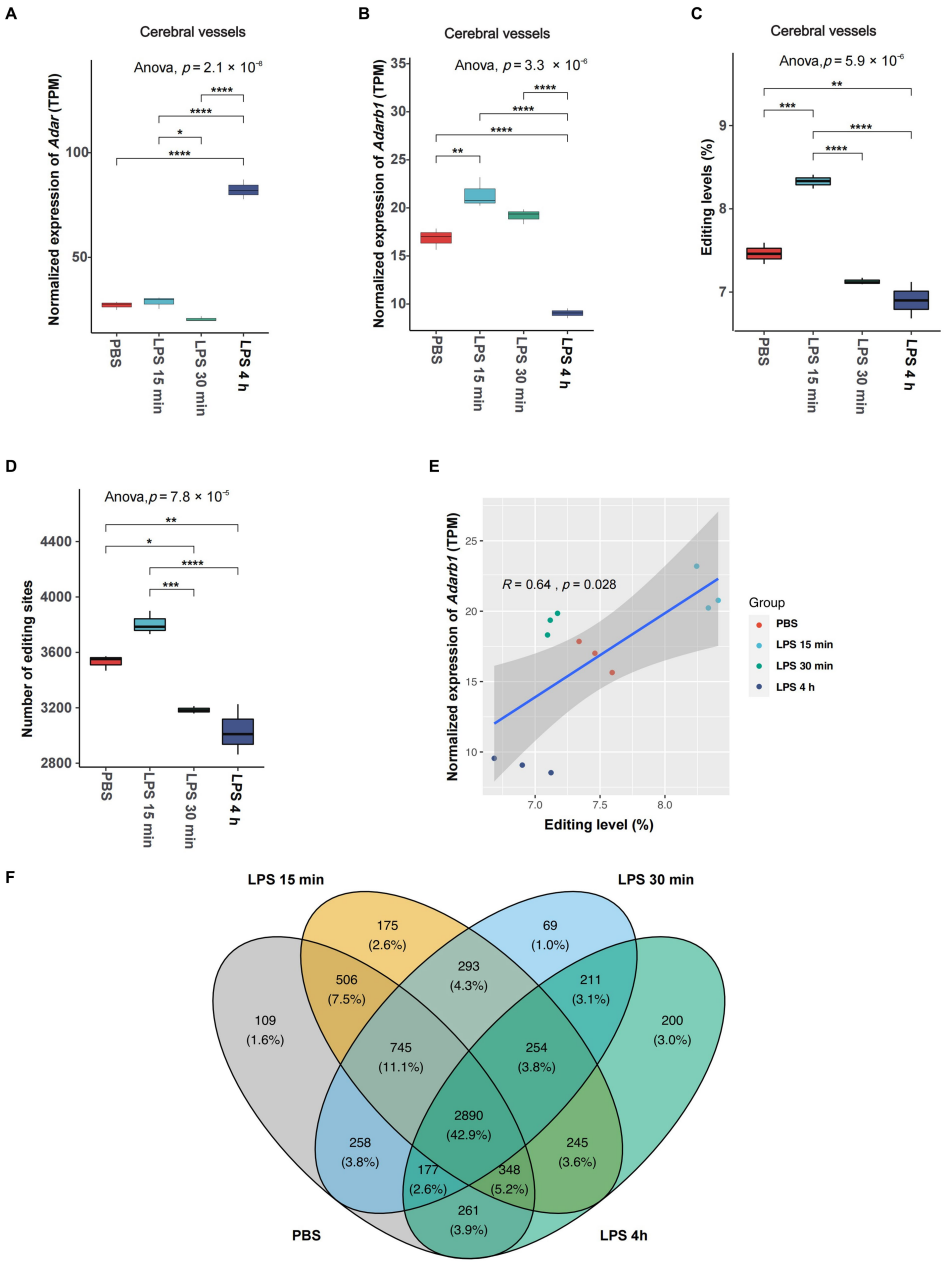
Overall changes in A-to-I RNA editing activities in cerebral vessels during acute neuroinflammation. **(A,B)** Expression of editing enzyme *Adar* and *Adarb1* in cerebral vessels. **(C)** The average A-to-I RNA editing level and **(D)** number of editing events in cerebral vessels are shown. **(E)** Spearman correlation between the relative expression level and the avarge A-to-I RNA editing level in cerebral vessels. **(F)** Venn plot comparing A-to-I editing events detected in cerebral vessels among groups. *, *p* < 0.05; **, *p* < 0.01; ***, *p* < 0.001; ****, *p* < 0.0001.

We first looked into the expression of RNA editing enzymes, including *Adar* and *Adarb1*, in the SAE mouse model. The most profound expression changes in ADARs during acute neuroinflammation were found in cerebral vessels (Figures 1A,B). In cerebral vessels, *Adar* expression slightly decreased at 30 min but dramatically increased at 4 h after LPS treatment, whereas *Adarb1* expression rapidly increased at 15 min, and decreased at 4 h after LPS treatment. After LPS treatment, the average A-to-I RNA editing levels and the number of observed editing events in cerebral vessels increased rapidly at 15 min, followed by a substantial decrease at 30 min and 4 h (Figures 1C,D). Spearman correlation analysis revealed a significant correlation between the average RNA editing level and *Adarb1* expression (Figure 1E).

Although the expressions of *Adar* and *Adarb1* or the number of observed editing events did not show significant changes in CECs during SAE (Supplementary Figure S1), the overall A-to-I RNA editing level significantly decreased 2 h after LPS treatment (Supplementary Figure S1C). Neither the expression of *Adar* and *Adarb1* nor the average RNA editing level show significant changes in microglia during acute neuroinflammation (Supplementary Figures S2A–C).

The existence of individual RNA editing events was then compared among different time point groups. The Venn plot in Figure 1F shows that only 43.2% of the RNA editing events were observed across all groups in cerebral vessels, pointing to the high dynamics of RNA editing during SAE. Similar results were also observed in CECs (Supplementary Figure S1E) and microglia (Supplementary Figure S2E).

Annotation of A-to-I RNA editing events showed a relatively stable composition of functional categories during acute inflammation in SAE (Supplementary Figure S3). The largest proportion of A-to-I RNA editing events was 3′-untranslated region (UTR) editing in cerebral vessels and microglia and missense editing in CECs (Supplementary Figure S3).

Functional enrichment analysis also indicated dynamic changes of A-to-I RNA editing during acute neuroinflammation in SAE by identifying differential gene functions and pathways significantly enriched by edited genes in some time points but not others. The most differentially enriched biological processes and pathways in cerebral vessels are shown in Supplementary Figures S4A,B, such as vascular endothelial growth factor (VEGF) and receptor signaling, MAPK signaling, Apelin signaling, FoxO signaling, PI3K−Akt signaling, spliceosome, neuron projection development, and bacterial invasion of epithelial cells. The most differentially enriched biological processes and pathways in CECs are shown in Supplementary Figures S4C,D, such as protein localization to phagophore assembly site, positive regulation of protein dephosphorylation, regulation of cell cycle, positive regulation of mRNA metabolic process, protein K63−linked deubiquitination, cytoplasmic pattern recognition receptor signaling, Kaposi sarcoma−associated herpesvirus infection, B cell and T cell receptor signaling, and VEGF signaling. The most differentially enriched biological processes and pathways in microglia are shown in Supplementary Figures S4E,F, such as Huntington disease, pertussis, positive regulation of macroautophagy, cellular response to interleukin−6, mRNA catabolic process, Ras protein signal transduction, peroxisomal membrane transport, canonical Wnt signaling, protein K63−linked deubiquitination, mRNA surveillance, cytosolic DNA−sensing pathway, and RIG−I−like receptor signaling.

### Differential A-to-I RNA editing during acute neuroinflammation in SAE

**Figure 2:**
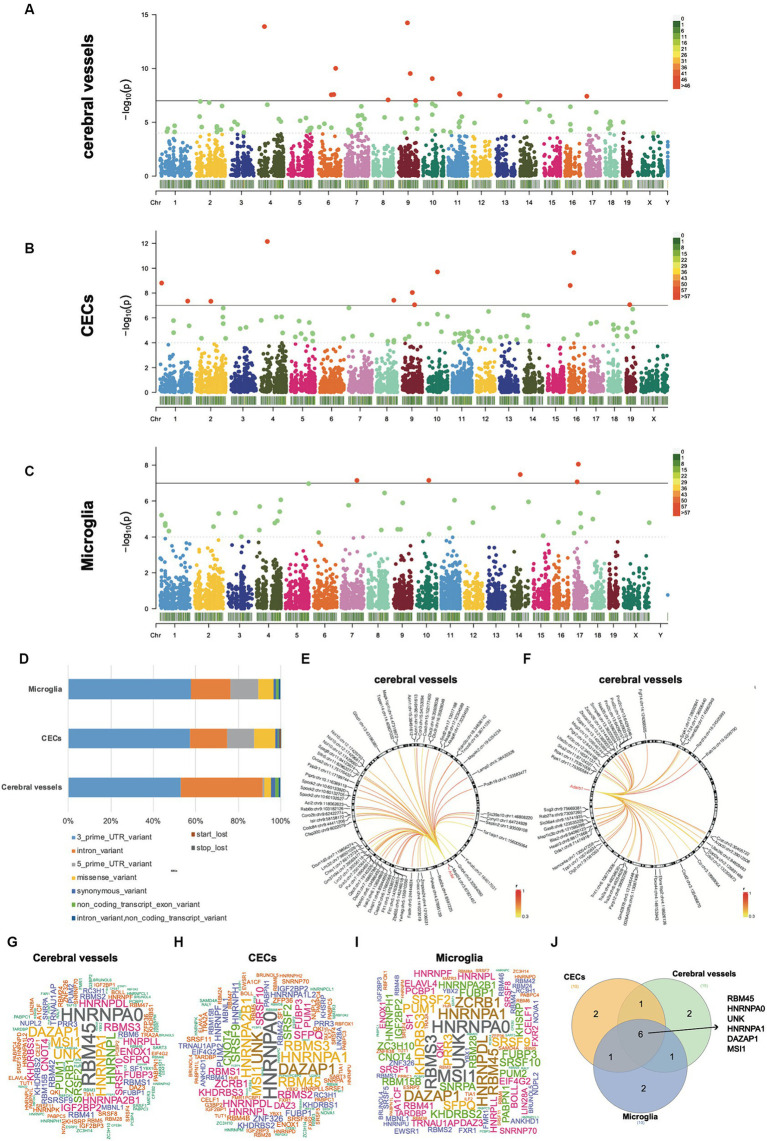
Differential A-to-I RNA editing during acute neuroinflammation. **(A–C)** Manhattan plots showing GLM *p*-values of individual A-to-I RNA editing events in cerebral vessels, CECs, and microglia. The solid horizontal line indicates *p* = 1.0 × 10^−7^, and the dashed horizontal line indicates *p* = 1.0 × 10^−4^. **(D)** Functional category distribution of DRE events in cerebral vessels, CECs, and microglia. **(E,F)** Differential RNA editing events associated with *Adar* and *Adarb1* expressions in cerebral vessels. **(G–I)** Wordcloud plots showing the frequency of RBPs with binding sites overlapping with DRE sites. **(J)** Venn plot showing six RBPs shared by the top 10 frequent RBPs in the cerebral vessels, CECs, and microglia shown in panels **(G–I)**. GLM, generalized linear model; DRE, differential RNA editing; RBPs, RNA binding proteins; CECs, cerebral endothelial cells.

To identify the changes in RNA editing associated with acute neuroinflammation during SAE, the RNA editing levels of the sites among different time point groups were compared using the GLM method. 371 events in 295 genes in cerebral vessels (Supplementary Tables S1, S2), 355 events in 319 genes in CECs (Supplementary Tables S3, S4), and 85 events in 83 genes in microglia (Supplementary Tables S5, S6) showed DRE (Figures 2A–C). Importantly, most of the differentially edited genes showed differential expression during acute neuroinflammation, suggesting the potential effects of DRE on gene expression (Supplementary Tables S2, S4, S6). Moreover, the largest proportion of functional categories of these DRE events were 3′-UTR in cerebral vessels, CECs, and microglia, with a much higher proportion of intronic DRE in cerebral vessels than in CECs and microglia, as shown in Figure 2D. Notably, many DRE events in cerebral vessels were positively correlated with *Adar* and *Adarb1* expressions (Figures 2E,F and Supplementary Tables S7, S8). PCA based on these DRE events showed that the samples of different time point groups could be well separated, with a large contribution rate of PC1 (cerebral vessels: 46.85%; CECs, 32.78%; and microglia, 33.03%, respectively) (Supplementary Figure S5).

Notably, several missense DRE events showed a more than 5% intergroup difference in editing levels during acute neuroinflammation (Table 1). Seven of these missense DRE events were found in cerebral vessels, including *Azin1* p.S367G, *Tmem63b* p.Q619R, *Grik5* p.K889R, *Zfp771* p.K220R, *Ube2o* p.S93G, and *Cacng8* p.S251G. Twelve were found in CECs, including *Myo1e* p.Q1009R, *Sgpp1* p.T89A, *Dhx36* p.E945G, *Cdk13* p.Q103R and p.K96R, *Cyfip2* p.K320E, *Lrrc8b* p.Q554R, *Taf9b* p.E40G, *Plec* p.H3003R, *Ccni* p.R75G, *Akap1* p.S433G, and *Lemd2* p.S226G. Only one, *Tagap1* p.T187A, was found in Microglia. Notably, most of these differentially edited genes showed significantly differential expression (GLM FDR < 0.05) in the same tissue or cells during acute neuroinflammation. In addition, DRE was also observed in lincRNAs, such as maternally expressed gene 3 (*Meg3*) in cerebral vessels, *AW112010* in CECs, and macrophage M2 polarization regulator (*Mm2pr*) in microglia.

**Table 1:** Differential missense A-to-I editing with at least 5% intergroup editing level difference in cerebral vessels, CECs, and microglia during acute neuroinflammation.

Gene symbol	Amino acid change	cDNA change	p.GLM	Full name	SIFT prediction	Average RNA editing level			
						PBS	LPS 15 min	LPS 30 min	LPS 4 h
Cerebral vessels									
Azin1*	p.S367G	c.1099A > G	2.50 × 10^−6^	Antizyme inhibitor 1	Tolerated	17.3	9	6.6	19.1
Tmem63b*	p.Q619R^#^	c.1856A > G	0.0016	Transmembrane protein 63b	Deleterious	25.9	41.4	26.5	27.8
Grik5*	p.K889R	c.2666A > G	9.43 × 10^−5^	Glutamate receptor, ionotropic, kainate 5 (gamma 2)	Tolerated low confidence	15.5	17.7	16.3	22.7
Zfp771*	p.K220R	c.659A > G	0.0046	Zinc finger protein 771	Tolerated	7.9	16.7	17.4	19.4
Ube2o*	p.S93G	c.277A > G	0.0002	Ubiquitin-conjugating enzyme E2O	Tolerated	26.2	52.9	29.7	10.2
Cacng8*	p.S251G^#^	c.751A > G	0.0013	Calcium channel, voltage-dependent, gamma subunit 8	Deleterious	17.4	12.3	16	24.5
Dact3*	p.K403E^#^	c.1207A > G	0.0019	Disheveled-binding antagonist of beta-catenin 3	Deleterious	1.4	7.2	2.4	10.3
CECs									
*Myo1e*	p.Q1009R	c.3026A > G	0.0019	Myosin IE	Tolerated	2	10.3	3.3	2.4
Sgpp1*	p.T89A	c.265A > G	3.03 × 10^−7^	Sphingosine-1-phosphate phosphatase 1	Tolerated	0.7	7.6	0	0
Dhx36*	p.E945G^#^	c.2834A > G	7.37 × 10^−6^	DEAH (Asp-Glu-Ala-His) box polypeptide 36	Deleterious	0	6.1	0	0
Cdk13*	p.Q103R	c.308A > G	0.0002	Cyclin-dependent kinase 13	Tolerated low confidence	84.2	86.5	78.3	95.8
*Cyfip2*	p.K320E	c.958A > G	0.0002	Cytoplasmic FMR1 interacting protein 2	Tolerated	12.1	61.1	NA	NA
*Lrrc8b*	p.Q554R	c.1661A > G	0.0002	Leucine rich repeat containing 8 family, member B	Tolerated	17.8	3.4	13	10.7
Taf9b*	p.E40G^#^	c.119A > G	0.002	TATA-box binding protein associated factor 9B	Deleterious low confidence	21	5.2	2.2	0
*Plec*	p.H3003R	c.9008A > G	0.0003	Plectin	Tolerated	0	0	1.2	5.6
Ccni*	p.R75G^#^	c.223A > G	0.0062	Cyclin I	Deleterious	7.8	11.2	13.1	9.2
Akap1*	p.S433G	c.1297A > G	0.0009	A kinase (PRKA) anchor protein 1	Tolerated	1	9.4	0.9	0
Lemd2*	p.S226G^#^	c.676A > G	0.0013	LEM domain containing 2	Deleterious	0	1.3	5	1
Cdk13*	p.K96R	c.287A > G	0.0072	Cyclin-dependent kinase 13	Tolerated low confidence	13.4	34	29.8	31.3
Microglia									
Tagap1*	p.T187A	c.559A > G	6.10 × 10^−5^	T cell activation GTPase activating protein 1	Tolerated	3	1	6.1	15.9

*Genes that showed significantly differential expression with a GLM FDR < 0.05. ^#^Predicted to be deleterious by SIFT.

The RBPmap tool was then used to predict how DRE events overlapped with and potentially affected RBP binding sites (Figures 2G–J). Our results showed that six RBPs, including RBM45, HNRNPA0, UNK, HNRNPA1, DAZAP1, and MSI1, were shared among the top 10 frequent RBPs with binding sites potentially affected by DRE. Interestingly, all these six RBP genes showed significant differential expression in cerebral vessels with empirical GLM *p* < 0.05. *Dazap1*, *Hnrnpa0*, and *Unk* were also differentially expressed in CECs, whereas only *Hnrnpa1* was differentially expressed in microglia with empirical GLM *p* < 0.05 (Supplementary Table S9).

**Figure 3:**
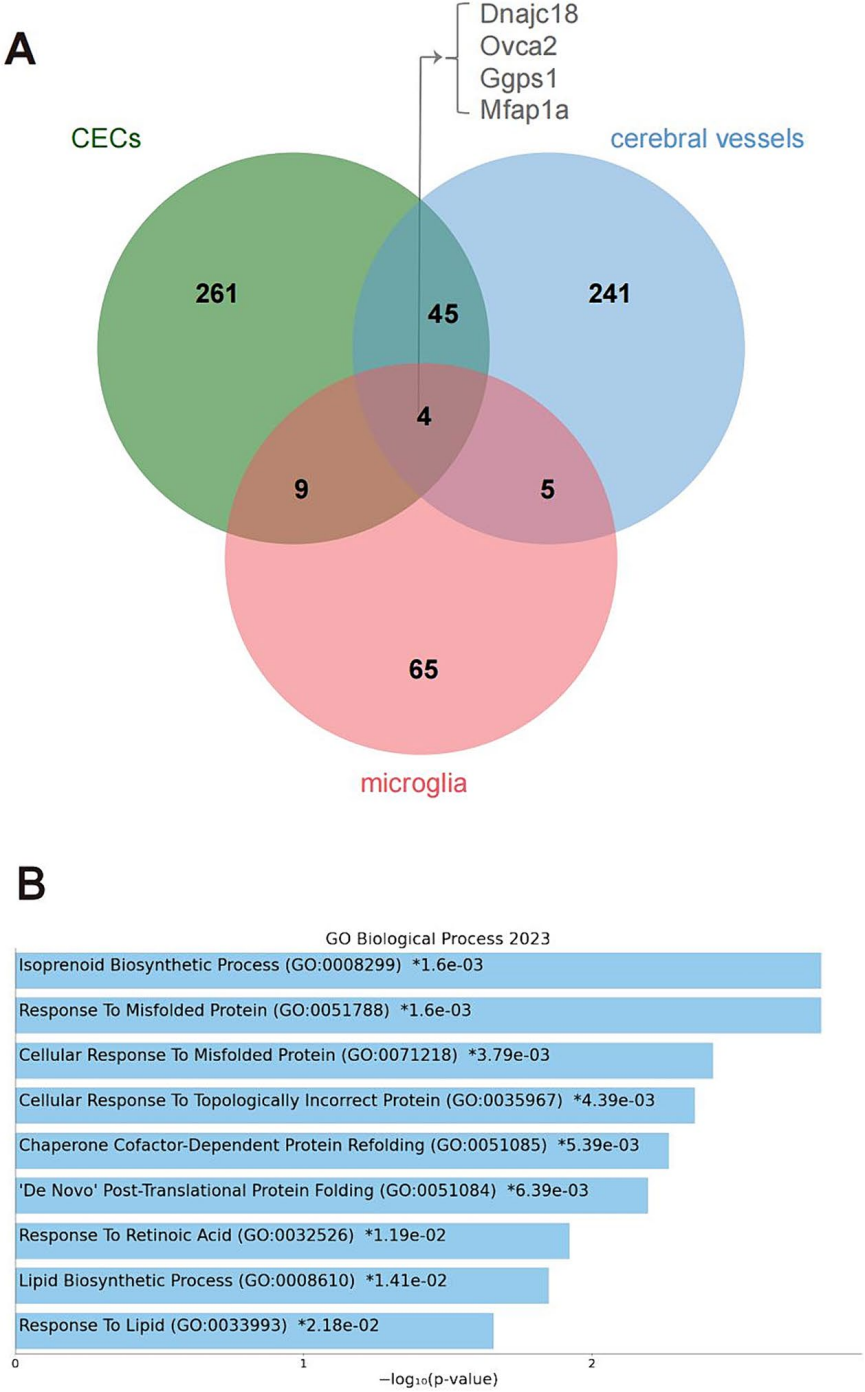
Genes differentially edited across cerebral vessels, CECs, and microglia during acute neuroinflammation. **(A)** Venn plot showing four common differentially edited genes in the tree tissues or cell types. **(B)** GO analysis showing biological processes significantly enriched by the four common differentially edited genes. CECs, cerebral endothelial cells; GO, gene ontology.

Four genes were differentially edited across cerebral vessels, CECs, and microglia (Figure 3A), including DnaJ Heat Shock Protein Family (Hsp40) member C18 (*Dnajc18*), OVCA2 Serine Hydrolase Domain Containing (*Ovca2*), Geranylgeranyl Diphosphate Synthase 1 (*Ggps1*) and Microfibril Associated Protein 1a (*Mfap1a*). Notably, 49 genes were differentially edited in both cerebral vessels and CECs, much more than those in both cerebral vessels (nine) or CECs (13) and microglia, pointing to a larger similarity of DRE between cerebral vessels and CECs. Gene ontology enrichment analysis further showed that these genes edited in both cerebral vessels and CECs were mainly involved in biological processes related to isoprenoid biosynthesis and response to misfolded protein and retinoic acid (Figure 3B).

### Time-dependent DRE during acute neuroinflammation

**Figure 4:**
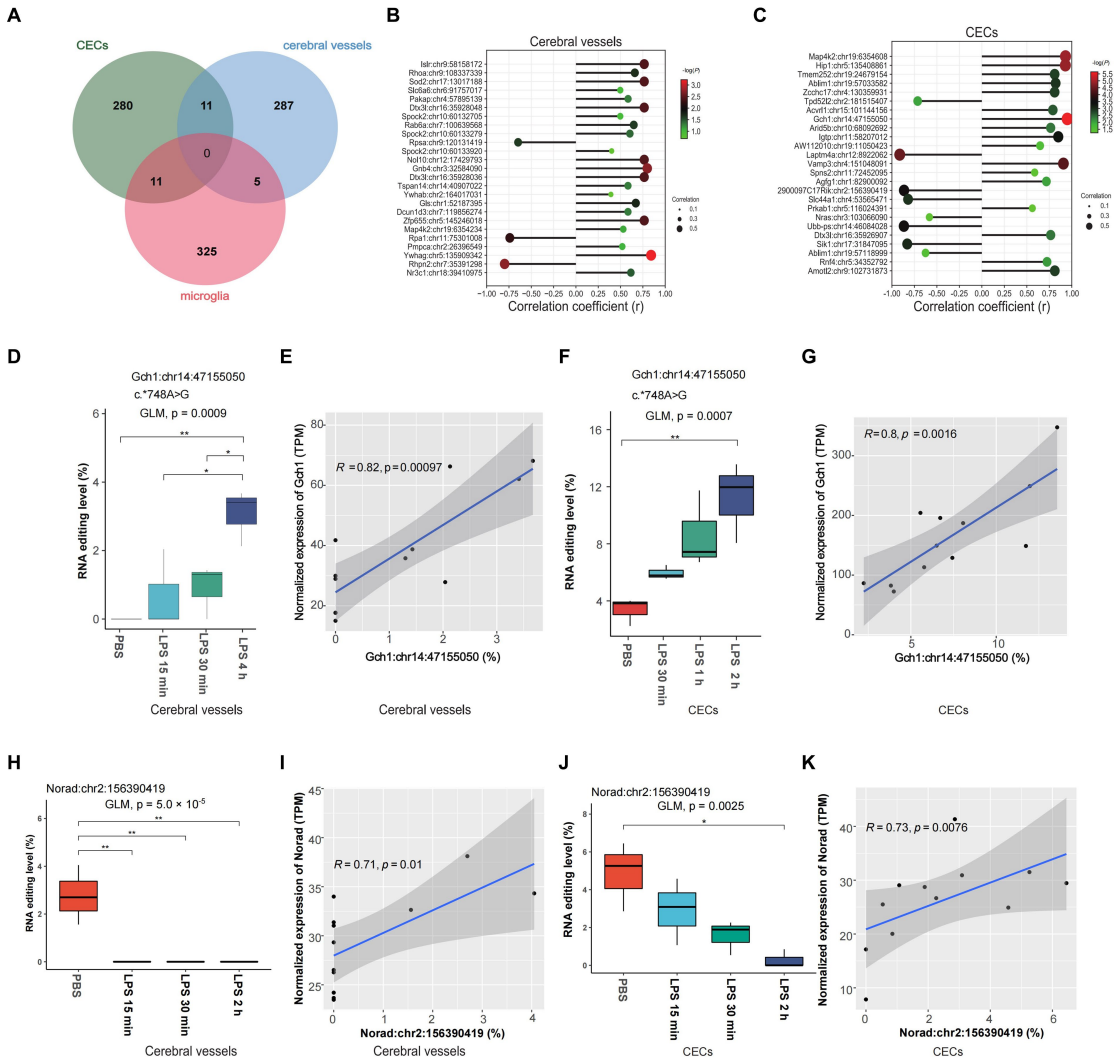
Time-dependent A-to-I RNA editing in cerebral vessels, CECs, and microglia during acute neuroinflammation. **(A)** Venn plot comparing the A-to-I editing events that highly correlated with time after LPS treatment (Spearman *p* < 0.05) in cerebral vessels, CECs, and microglia. **(B,C)** Spearman correlation of the top 25 differentially edited events strongly correlated with time in CECs and cerebral vessels. **(D)** RNA editing of *Gch1*:chr14:47155050 in cerebral vessels. **(E)** Spearman correlation between the editing level of *Gch1*:chr14:47155050 and *Gch1* expression in cerebral vessels. **(F)** RNA editing of *Gch1*:chr14:47155050 in CECs. **(G)** Spearman correlation between the editing level of *Gch1*:chr14:47155050 and *Gch1* expression in CECs. **(H)** RNA editing level of *Norad*:chr2:156390419 in cerebral vessels. **(I)** Correlation between levels of *Norad*:chr2:156390419 editing and *Norad* expression in cerebral vessels. **(J)** RNA editing level of *Norad*:chr2:156390419 in CECs. **(K)** The correlation between editing efficiency at *Norad*:chr2:156390419 editing and *Norad* expression in CECs. CECs, cerebral endothelial cells; *Gch1*, GTP cyclohydrolase1; *Norad, non-coding RNA actived by DNA damage.*

Spearman correlation analysis was used to identify time-dependent DRE events using time after LPS treatment as an independent continuous variable. Our results showed 304, 302, and 340 time-dependent DRE events in cerebral vessels (Supplementary Table S10), CECs (Supplementary Table S11), and microglia (Supplementary Table S12), respectively (all Spearman *p* < 0.05). Moreover, 11 time-dependent DRE events were shared by cerebral vessels and CECs, 11 were shared by CECs and microglia, and 5 were shared by cerebral vessels and microglia (Figure 4A). Among these time-dependent DRE events shared by cerebral vessels and CECs, two showed cis-regulatory effects on the edited gene expression, including 3′-UTR editing in GTP cyclohydrolase1 (*Gch1*) *Gch1*:chr14:47155050 and lincRNA editing in non-coding RNA activated by DNA damage (*Norad*) (*Norad*:chr2:156390419) (Figures 4D–K and Supplementary Tables S2, S4). The top 25 time-dependent events correlated with time in CECs and cerebral vessels, as shown in Figures 4B,C, most of which positively correlated with the edited gene expression. Notably, the correlation coefficient (*r*) was 0.95 and 0.81 for *Gch1*:chr14:47155050 in cerebral vessels (Figure 4E) and CECs (Figure 4G), respectively. Notably, the *Gch1* 3′-UTR RNA editing was observed only after LPS treatment but not in PBS controls. RBPmap prediction showed that *Gch1*:chr14:47155050 might affect the binding of PCBP to *Gch1* mRNA (Supplementary Figure S6), an RBP highly expressed in the nervous system. Both the editing and expression levels of *Norad* decreased in cerebral vessels (Figures 4H,I) and CECs (Figures 4J,K), respectively.

## Discussion

Our previous study implicated the association of brain A-to-I RNA editing with sepsis, yet its role in SAE remains to be further investigated. Through transcriptome-wide analyses, our study investigated the temporal dynamics of A-to-I RNA editing in cerebral vessels, CECs, and microglia during acute neuroinflammation in septic mice, underlining the importance of such epigenetic changes in the disease.

Existing studies have shown widespread A-to-I RNA editing among genes expressed in the CNS (Behm and Öhman, 2016), which could play potentially important roles in neurodevelopment and brain function. Inflammation and immune Moreover, A-to-I RNA editing was also associated with the pathogenesis of neurological and psychiatric disorders, including amyotrophic lateral sclerosis, epilepsy, developmental epileptic encephalopathy, depression, and schizophrenia (Yang et al., 2021). In line with such a role, our current study highlighted the role of A-to-I RNA editing during acute neuroinflammation in SAE. Neuroinflammation, vascular dysfunction, and BBB disruption were thought to play an important role in the progression of SAE (Kikuchi et al., 2019). Activation of CECs resulted in compromised BBB and brain dysfunction. Microglia also contributed to neuroinflammation. In this study, dynamic A-to-I RNA editing changes observed in different tissues or cell types might contribute to an in-depth understanding of the pathogenesis during acute neuroinflammation in SAE.

Notably, functional enrichment analysis suggested the involvement of A-to-I RNA editing in common important gene functions and pathways dynamically altered during acute neuroinflammation in SAE. The most commonly altered biological functions and pathways were related to inflammation and immune response, in line with the neuroinflammatory characteristic of SAE. Two of the common pathways that showed changes between cerebral vessels and CECs were related to VEGF and VEGFR signaling pathway and MAPK signaling pathway. The VEGF family and VEGFRs play a crucial role in the health and function of cerebral vessels and their endothelial cells. Studies suggest VEGF signaling through VEGFR2 might have neuroprotective effects, promoting neuronal survival under hypoxic conditions (Wittko-Schneider, et al., 2013; Silva-Hucha et al., 2021), and brain expression of *VEGFB* is associated with cognitive aging and Alzheimer’s disease (Mahoney et al., 2021).

Our analysis revealed numerous missense A-to-I RNA editing associated with acute neuroinflammation in various tissues or cell types, suggesting a potential role of A-to-I RNA editing in recoding proteins in SAE. As suggested by the SIFT prediction, these missense editing events could have functional impacts on the encoded protein. Notably, the differential *Azin1* S367G editing in cerebral vessels is conserved between humans and mice, which has been found to enhance cancer cell stemness, promote tumor angiogenesis, and might drive metastasis (Han et al., 2015; Wei et al., 2022). Our finding thus added to the biological functions of *Azin1* S367G editing by showing its involvement during acute neuroinflammation in SAE. In addition, our findings also suggested a potential involvement of lincRNA editing during acute neuroinflammation in SAE. *Meg3* could promote Nlrp3-mediated inflammation in microglia, and its elevated expression could induce endothelial dysfunction (Jiang et al., 2022). LncRNA *Aw112010* has recently been found to be a key modulator of inflammation. AW112010 could promote inflammatory T-cell differentiation by suppressing IL-10 expression through demethylation of H3K4, and its elevated expression might increase during monocyte aging (Barman et al., 2022). Intriguing, *Mm2pr*, a lincRNA differentially edited in microglia during acute neuroinflammation in our current study, has been recently identified as an essential modulator for M2 macrophage polarization with a potential role in macrophage-promoted tumorigenesis (Cao et al., 2019). Such differential lincRNA editing might modulate the acute neuroinflammation process in SAE, which warrants further investigation.

Our current study identified genes differentially edited across cerebral vessels, CECs, and microglia, which could be hotspots of A-to-I RNA editing during acute neuroinflammation. *Dnajc18* encodes a protein of the DNAJ family, which is highly expressed in the brain and plays a key role in neurodegenerative disorders, such as Parkinson’s Disease, and may also be involved in ubiquitin-dependent ERAD pathway and cellular response to misfolded protein (Huang et al., 2019; Zhang K. et al., 2022). In addition, *Dnajc18* has recently been identified to be involved in congenital and structural heart disorders and cardiomyopathy in both humans and mice (Spielmann et al., 2022). *Ovca2* encodes a serine hydrolase and is downregulated and degraded during retinoid-induced apoptosis (Prowse et al., 2002), with its role in SAE unknown. *Ggps1* encodes a protein belonging to the prenyltransferase family, and its knockout ameliorates ventilator-induced lung injury by regulating TLR2/4-AP-1 signaling (Wan et al., 2020). *Ggps1* is also involved in muscular dystrophy and promotes rab37-mediated autophagy (Tucker et al., 2020; Kaiyrzhanov et al., 2022; Wang et al., 2022).

In addition, our study revealed time-dependent dynamic changes in A-to-I RNA editing during acute neuroinflammation, especially *Gch1*: chr14:47155050. *Gch1* encodes a member of the GTP cyclohydrolase family, which could be upregulated in the vascular wall during inflammation and in endothelial cells stimulated by cytokines such as IL-6, hs-CRP, and LPS (Antoniades et al., 2011). Increased GCH1 expression leads to greater tetrahydrobiopterin (BH4) production, which acts as a defense mechanism against systemic inflammation by helping maximize eNOS function and maintaining endothelial function. Moreover, *GCH1* also plays a role in inflammatory and peripheral neuropathic pain (Nasser et al., 2013; Xiao et al., 2023). *Gch1* variants have recently been associated with the risk and age of onset of Parkinson’s disease (Pan et al., 2020). The impact of A-to-I RNA editing at *Gch1*:chr14:47155050 on SAE remained unanswered, but it could possibly be involved in BH4 production by regulating *Gch1* expression. In addition, *NORAD* in humans encodes a lincRNA reported to inhibit vascular endothelial cell senescence and apoptosis (Bian et al., 2020) and has a potential role in neurological diseases (Wang B. et al., 2020). The findings of time-dependent DRE in *Gch1* and *Norad* warranted further study on the role of RNA editing in cerebral vessels and CECs in SAE.

In conclusion, our current study demonstrates dynamic alterations in A-to-I RNA editing in cerebral vessels, CECs, and microglia in a mouse model during acute neuroinflammation in SAE. Therefore, our findings provided new insight into understanding the role played by A-to-I RNA editing in SAE.

## Data availability statement

Publicly available datasets were analyzed in this study. This data can be found here: https://www.ncbi.nlm.nih.gov/geo under accession IDs GSE155516 and GSE155517.

## Ethics statement

The requirement of ethical approval was waived by the Ethics Committee of Jiangnan University for the studies involving animals because the study was a reanalysis of publicly available datasets. The studies were conducted in accordance with the local legislation and institutional requirements.

## Author contributions

Y-NL: Data curation, Formal analysis, Writing – original draft. Y-PL: Formal analysis, Investigation, Methodology, Visualization, Writing – review & editing. J-QZ: Data curation, Formal analysis, Investigation, Visualization, Writing – original draft. NL: Conceptualization, Methodology, Writing – review & editing. Z-YW: Methodology, Software, Writing – review & editing. YR: Writing – review & editing. J-HC: Conceptualization, Data curation, Supervision, Writing – review & editing. Y-YJ: Formal analysis, Funding acquisition, Methodology, Supervision, Writing – review & editing.

## Footnotes

^1^ https://www.ncbi.nlm.nih.gov/geo/

^2^ https://www.ensembl.org/vep

^3^ http://srv00.recas.ba.infn.it/atlas/index.html

^4^ https://maayanlab.cloud/Enrichr/

## Supplementary material

SUPPLEMENTARY FIGURE S1 RNA editing activities in CECs during acute neuroinflammation. **(A,B)** Expression of editing enzyme *Adar* and *Adarb1* in CECs and **(C,E)** A-to-I RNA editing events identified from mouse CECs. **(C)** A-to-I RNA editing levels and **(D)** number of editing sites in cerebral vessels are shown. **(E)** Venn plot comparing the A-to-I editing sites detected in 2 or more samples among groups.

SUPPLEMENTARY FIGURE S2 RNA editing activities in microglia during acute neuroinflammation. **(A,B)** Expression of editing enzyme *Adar* and *Adarb1* in microglia and **(C–E)** A-to-I RNA editing events identified from mouse microglia. **(C)** A-to-I RNA editing levels and **(D)** number of editing sites in cerebral vessels are shown. **(E)** Venn plot comparing the A-to-I editing sites detected in 2 or more samples among groups.

SUPPLEMENTARY FIGURE S3 A-to-I RNA editing variants during acute neuroinflammation. Results are shown for **(A)** cerebral vessels, **(B)** CECs, and **(C)** microglia.

SUPPLEMENTARY FIGURE S4 The most differentially enriched biological processes and pathways during acute neuroinflammation. Results of GO biological processes and KEGG pathways are shown for **(A,B)** cerebral vessels, **(C,D)** CECs, and **(E,F)** microglia.

SUPPLEMENTARY FIGURE S5 Principle component analysis of the A-to-I RNA editing events differentially edited between different groups during acute neuroinflammation. Results are shown for **(A)** cerebral vessels, **(B)** CECs, and **(C)** microglia.

SUPPLEMENTARY FIGURE S6 The results of overlapping RBP binding sites of Gch1:chr14:47155050 using the RBPmap.
