# Supplementary figures and images for: Dynamic A-to-I RNA editing during acute neuroinflammation in sepsis-associated encephalopathy
Source: Front Neurosci. 2024 Aug 2;18:1435185. doi: 10.3389/fnins.2024.1435185 (PMC11328407; doi:10.3389/fnins.2024.1435185)

**A**

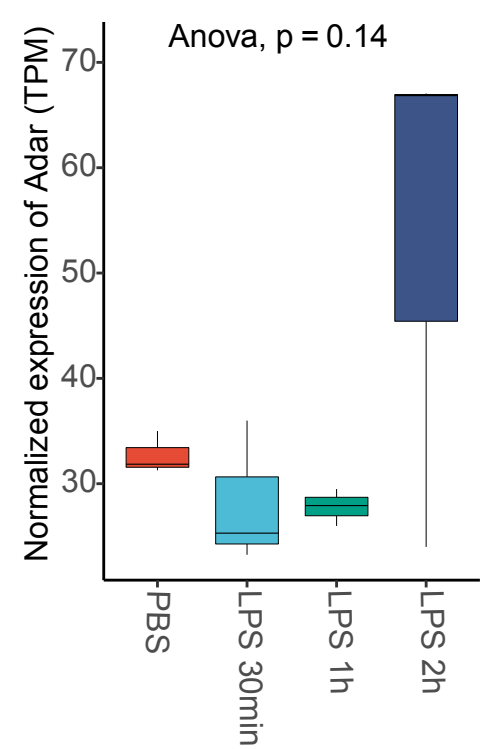

**B**

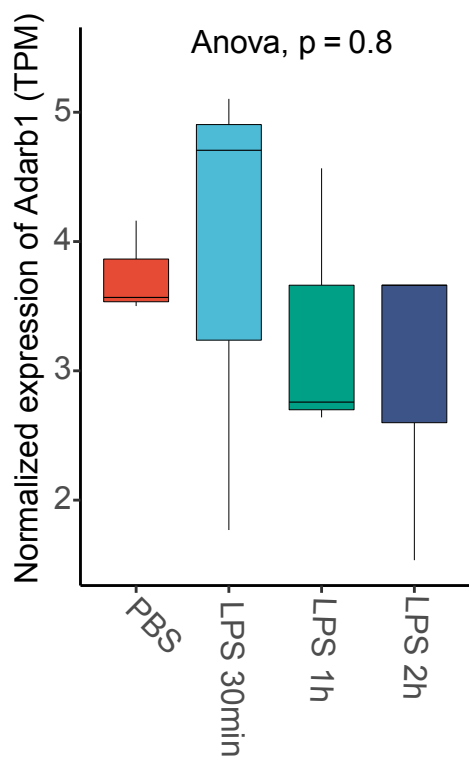

**C**

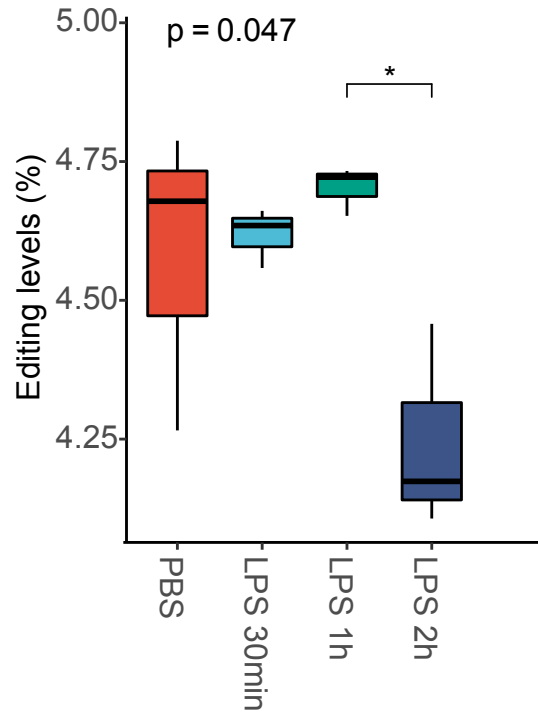

**D**

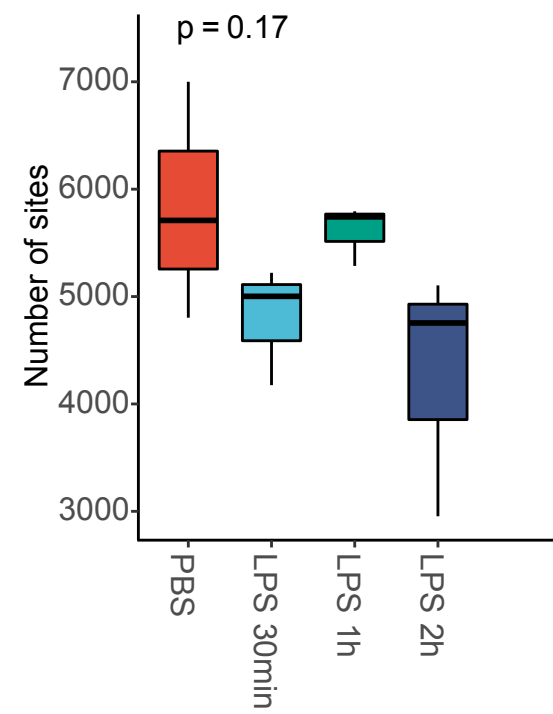

**E**

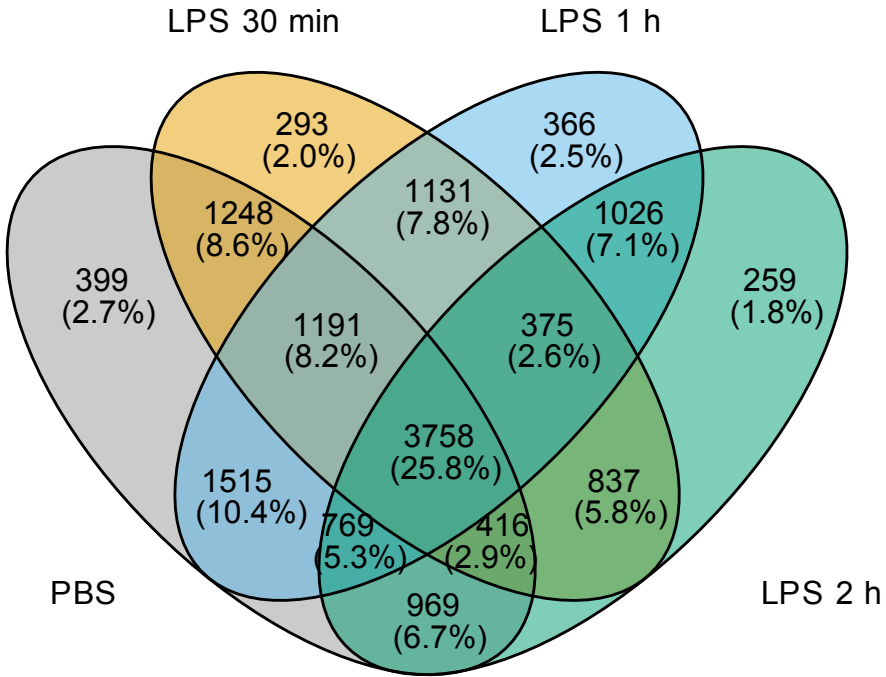

Supplement: SUPPLEMENTARY FIGURE S1 — RNA editing activities in CECs during acute neuroinflammation. (A,B) Expression of editing enzyme Adar and Adarb1 in CECs and (C,E) A-to-I RNA editing events identified from mouse CECs. (C) A-to-I RNA editing levels and (D) number of editing sites in cerebral vessels are shown. (E) Venn plot comparing the A-to-I editing sites detected in 2 or more samples among groups. [file Data_Sheet_1.PDF]

**A**

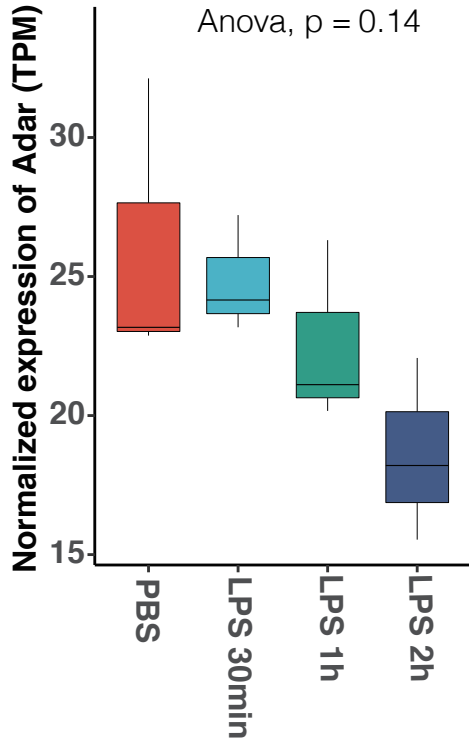

**B**

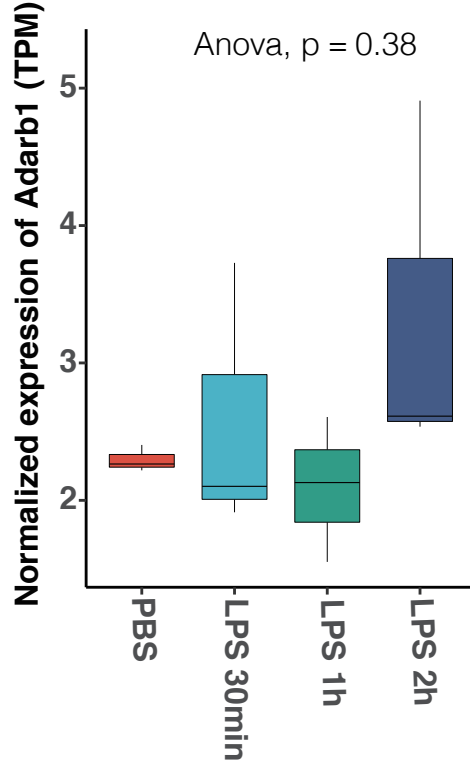

**C**

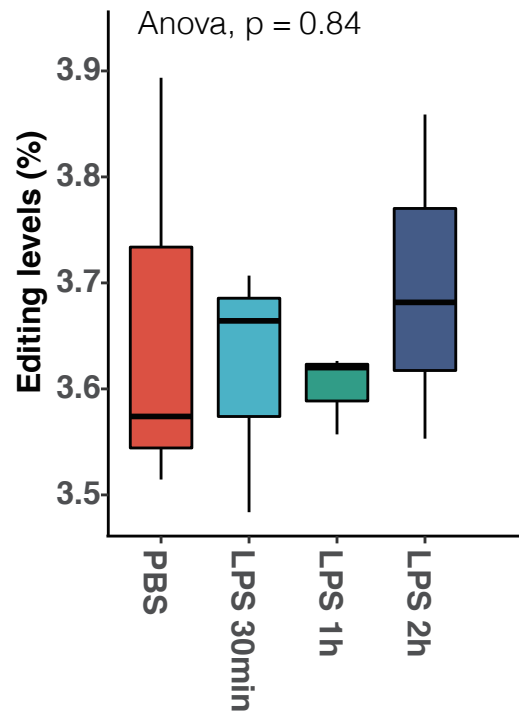

**D**

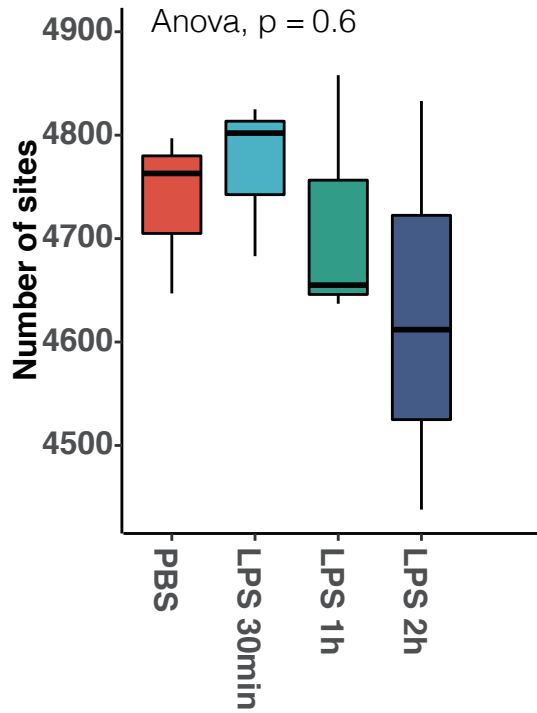

**E**

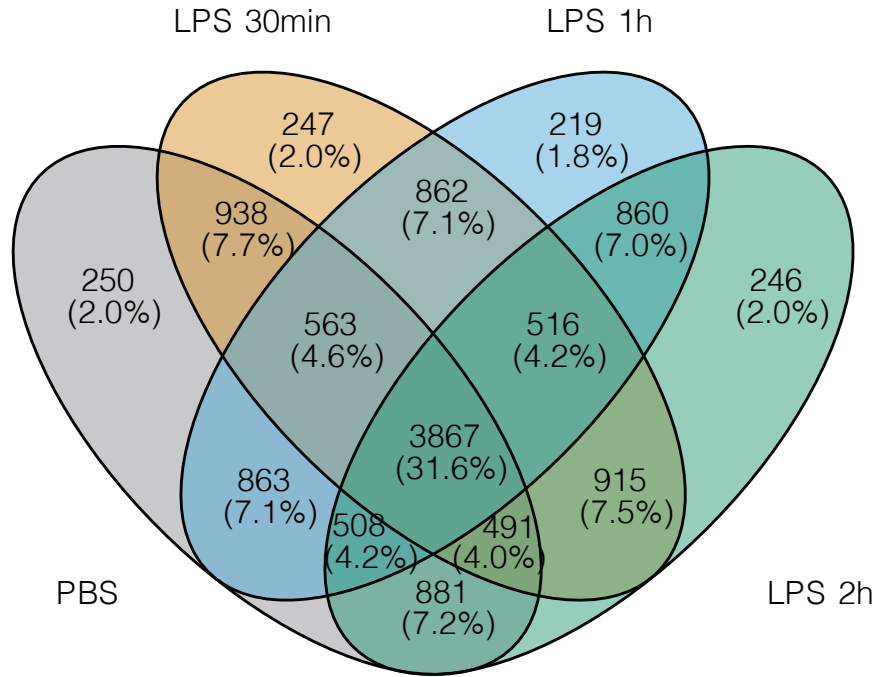

Supplement: SUPPLEMENTARY FIGURE S2 — RNA editing activities in microglia during acute neuroinflammation. (A,B) Expression of editing enzyme Adar and Adarb1 in microglia and (C–E) A-to-I RNA editing events identified from mouse microglia. (C) A-to-I RNA editing levels and (D) number of editing sites in cerebral vessels are shown. (E) Venn plot comparing the A-to-I editing sites detected in 2 or more samples among groups. [file Data_Sheet_2.PDF]

**A**

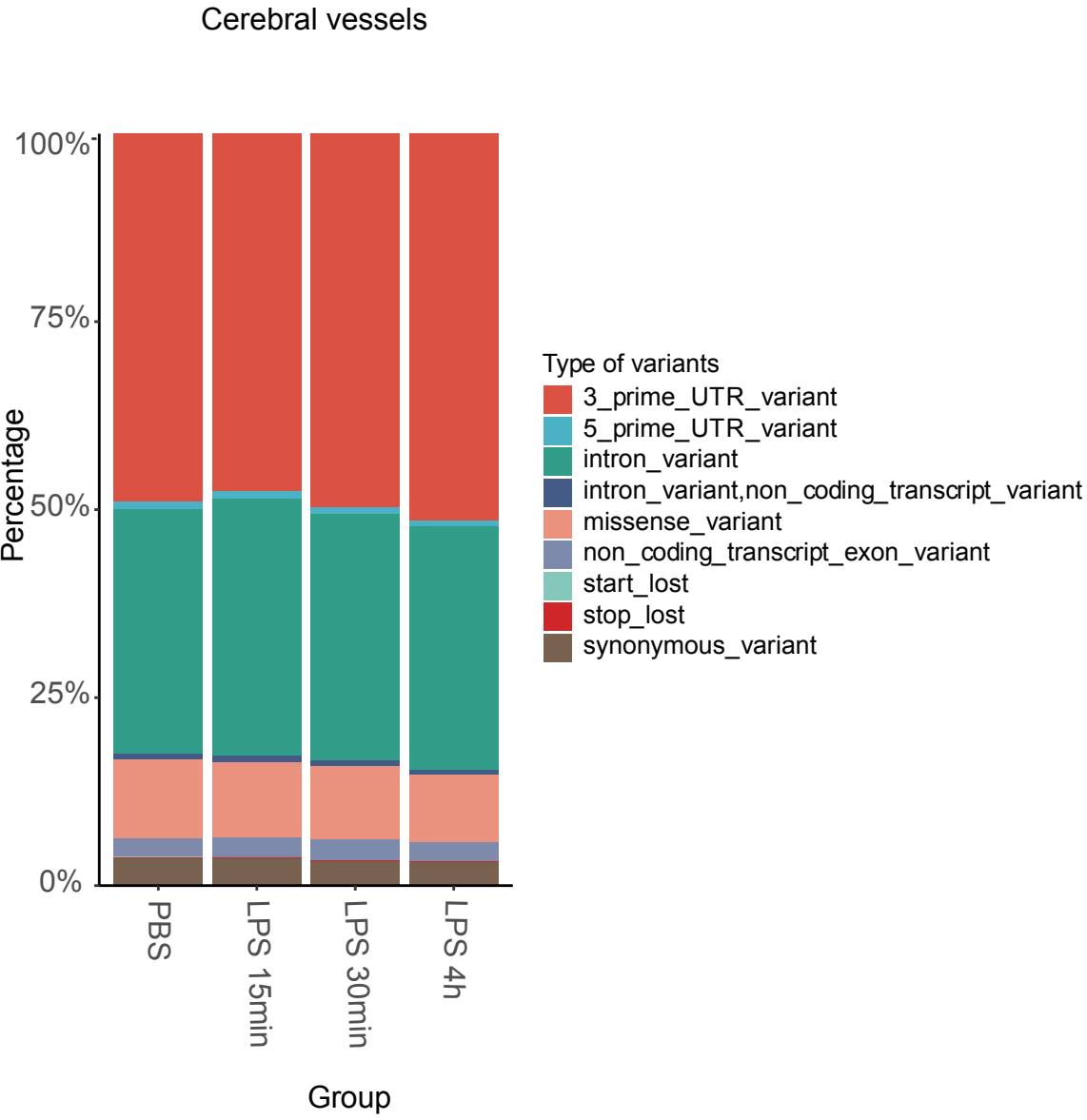

**B**

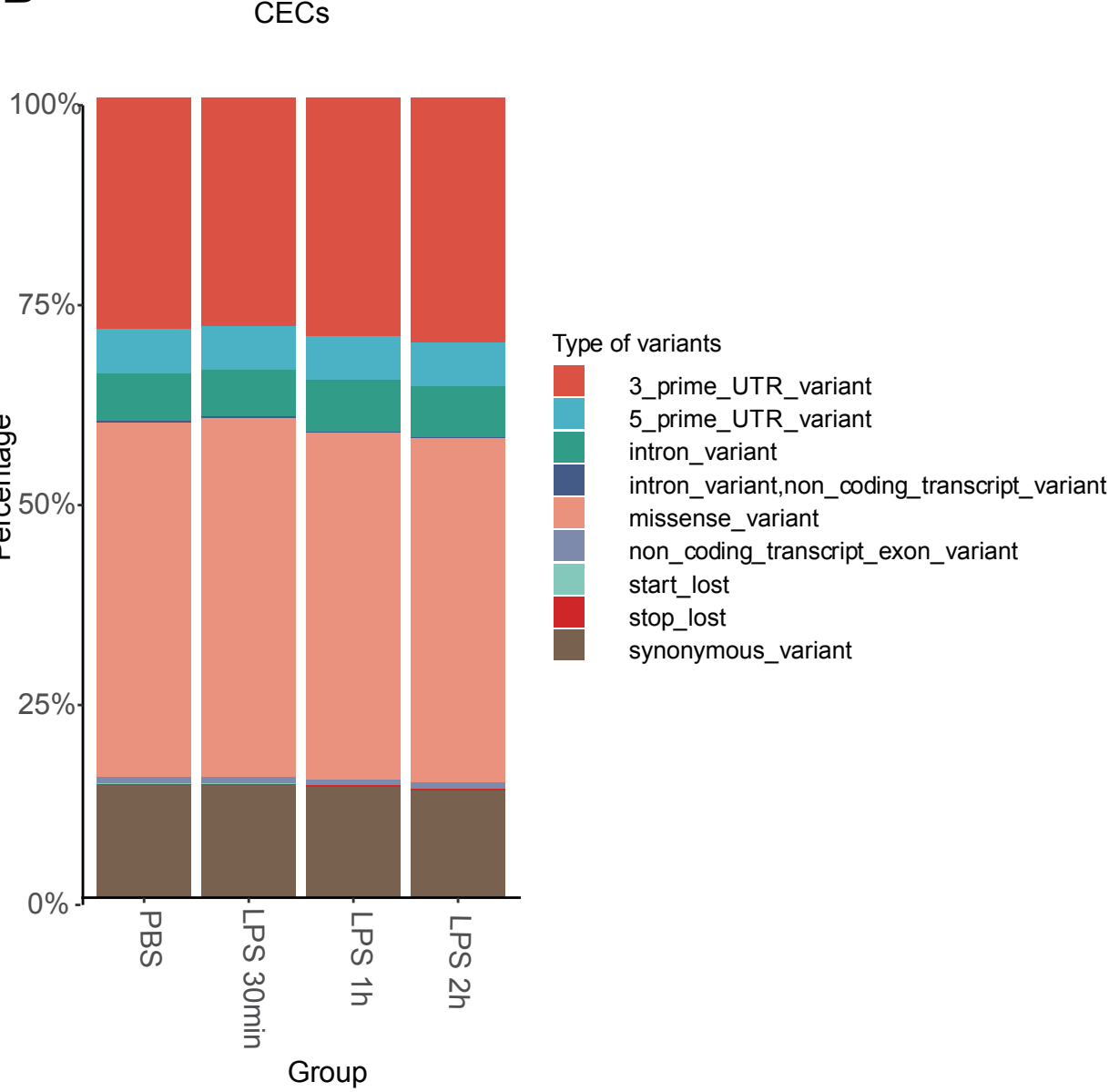

**C**

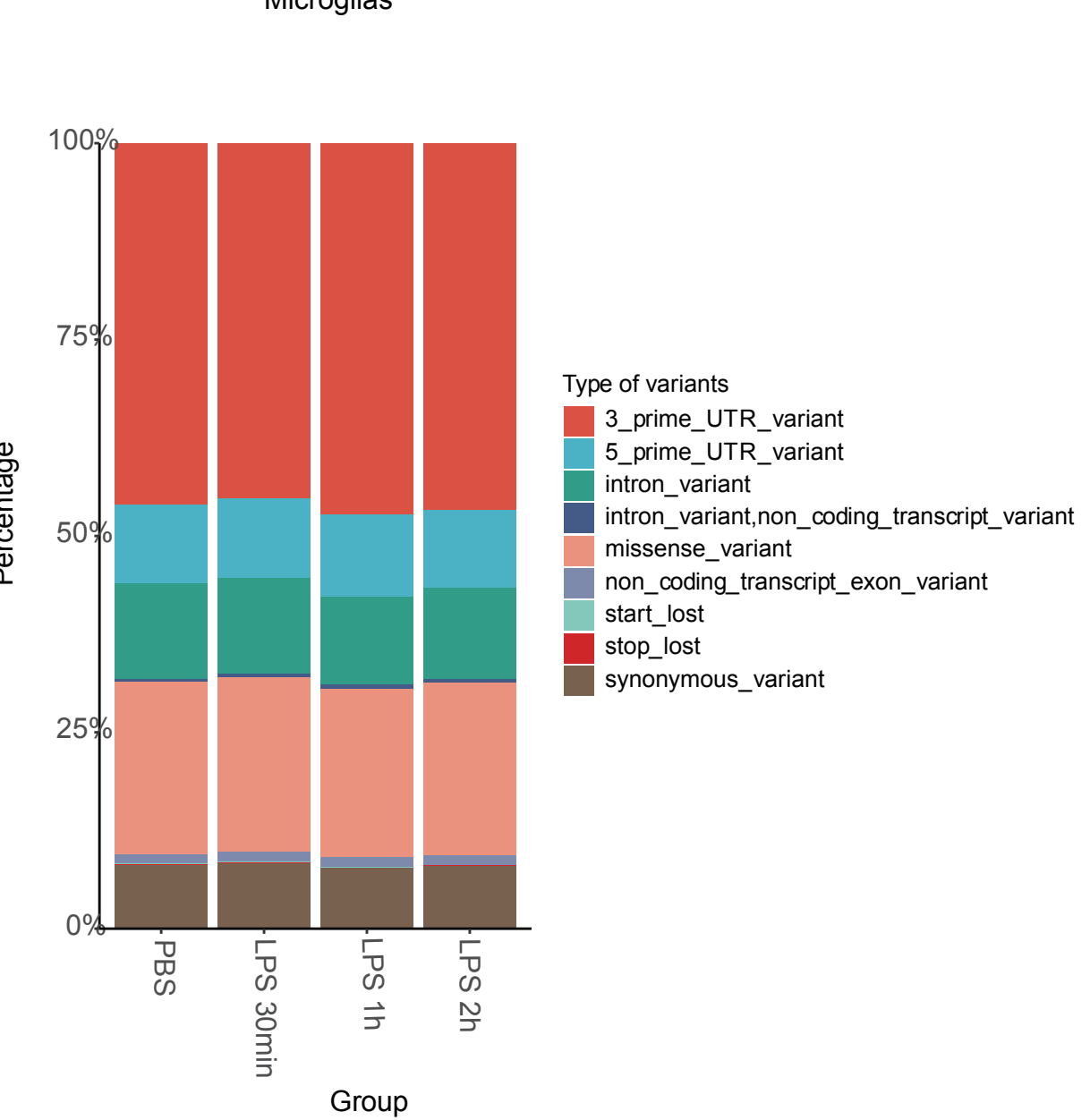

Supplement: SUPPLEMENTARY FIGURE S3 — A-to-I RNA editing variants during acute neuroinflammation. Results are shown for (A) cerebral vessels, (B) CECs, and (C) microglia. [file Data_Sheet_3.PDF]

**A**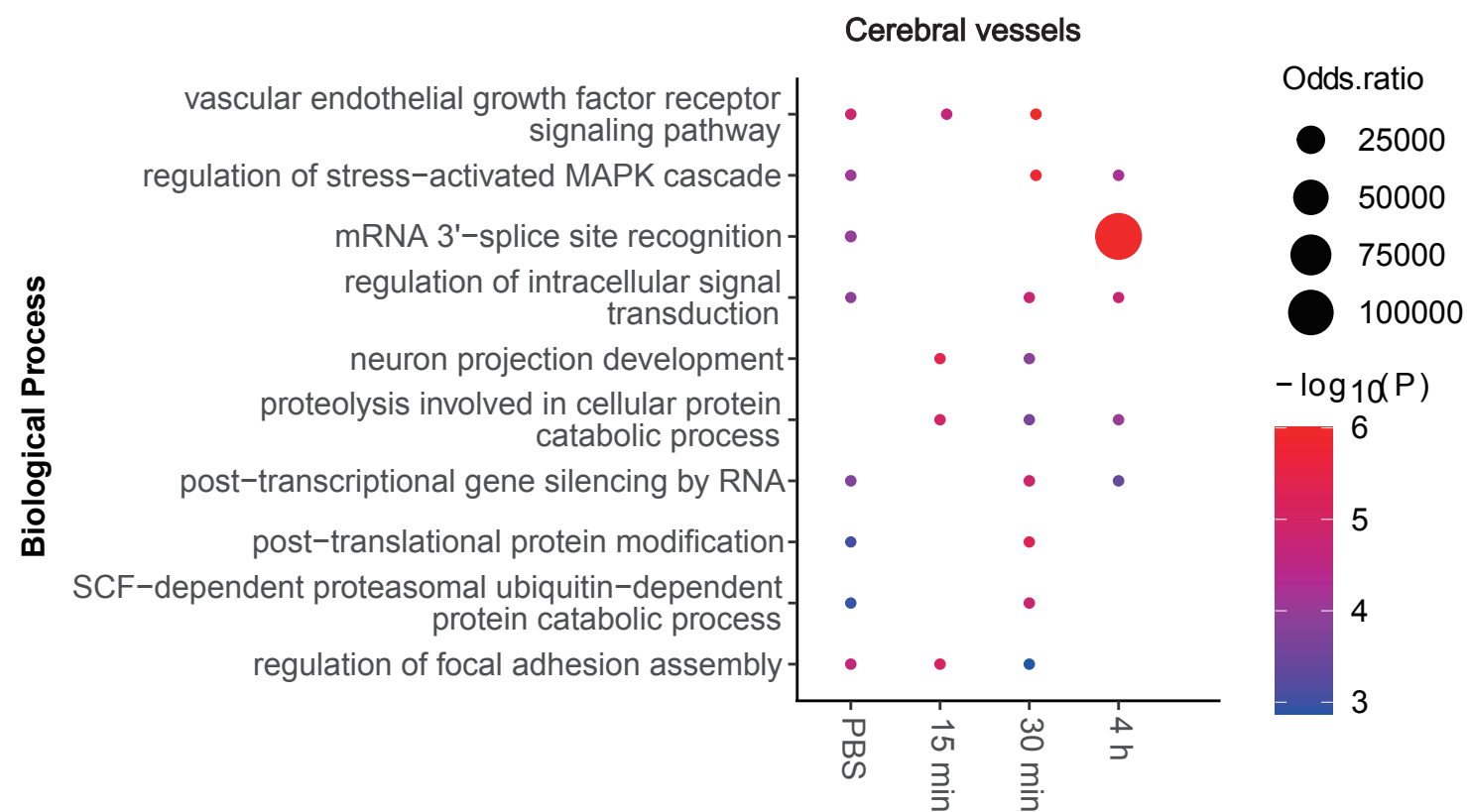**B**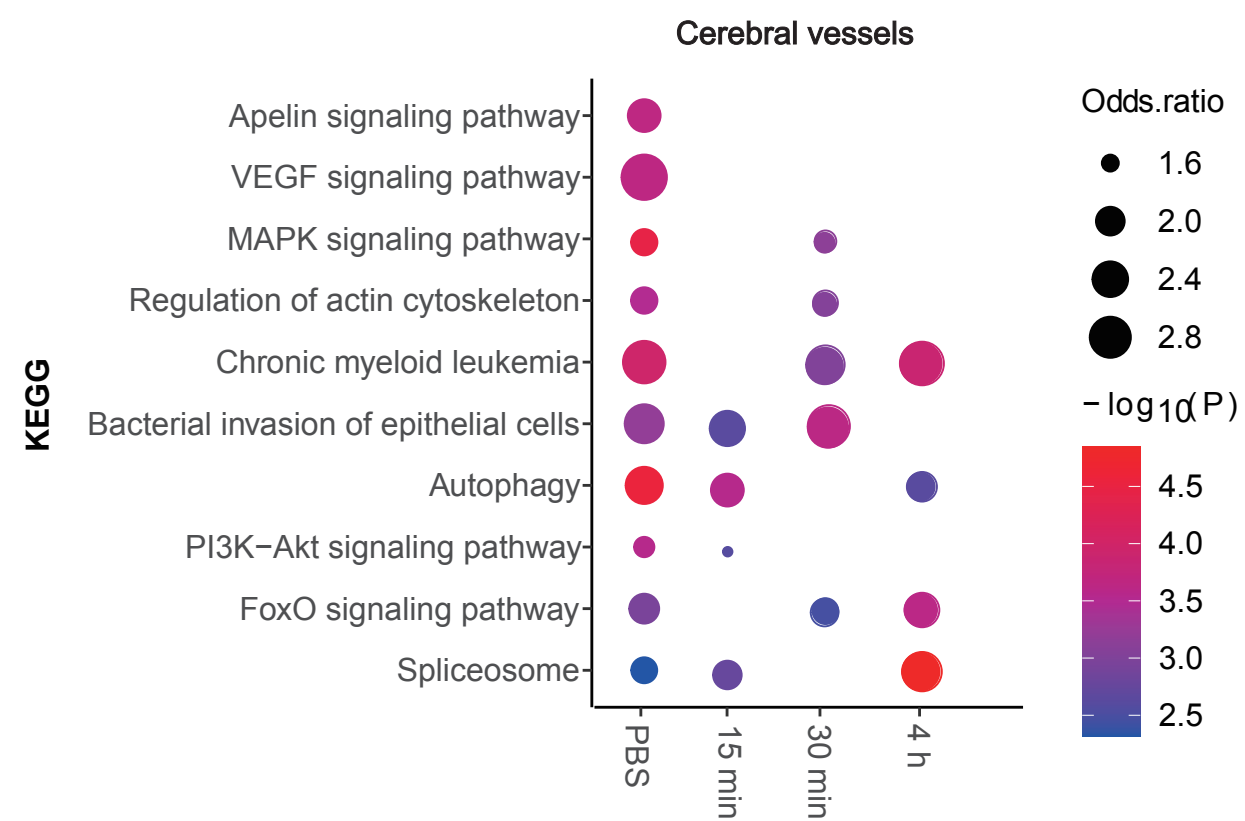**C**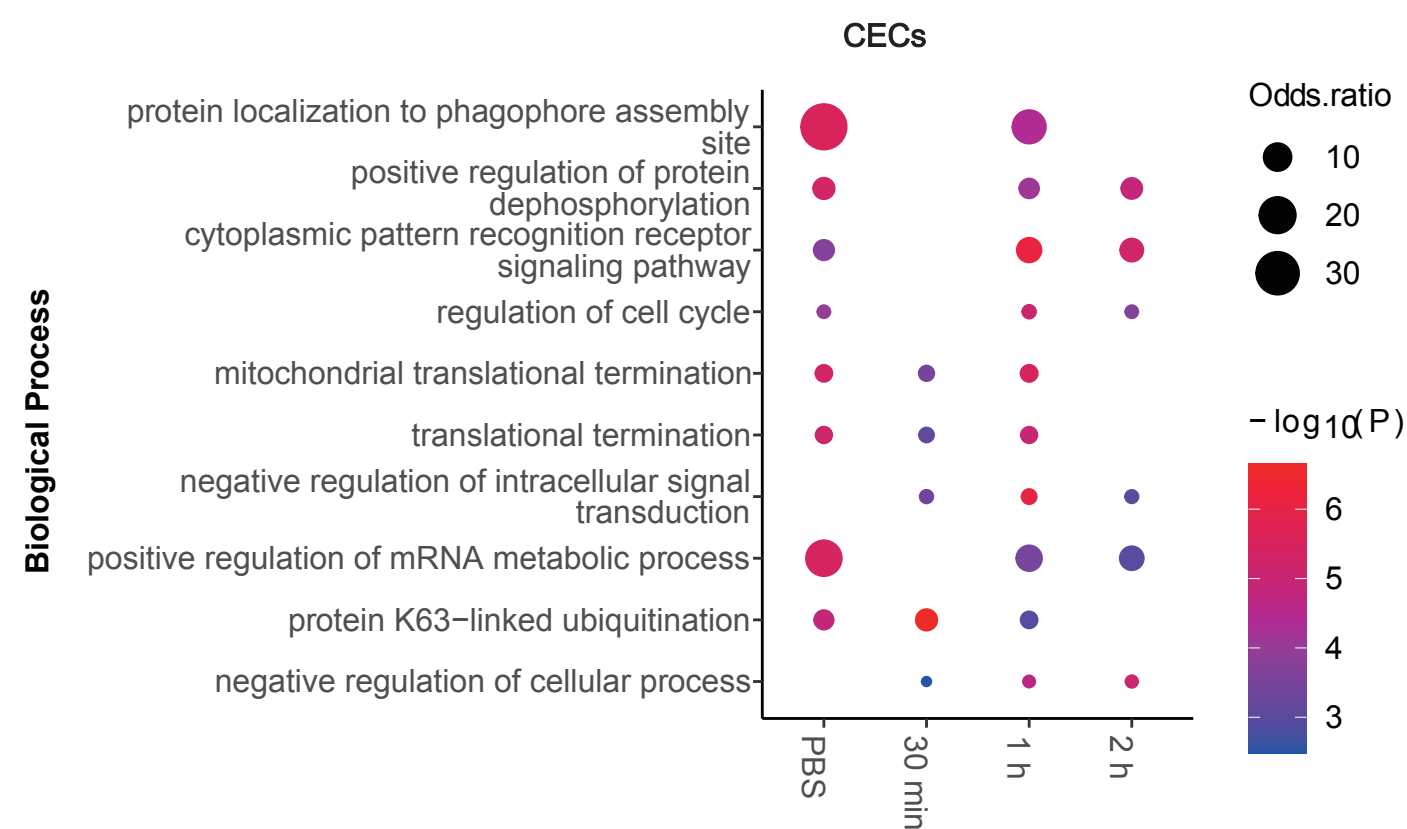**D**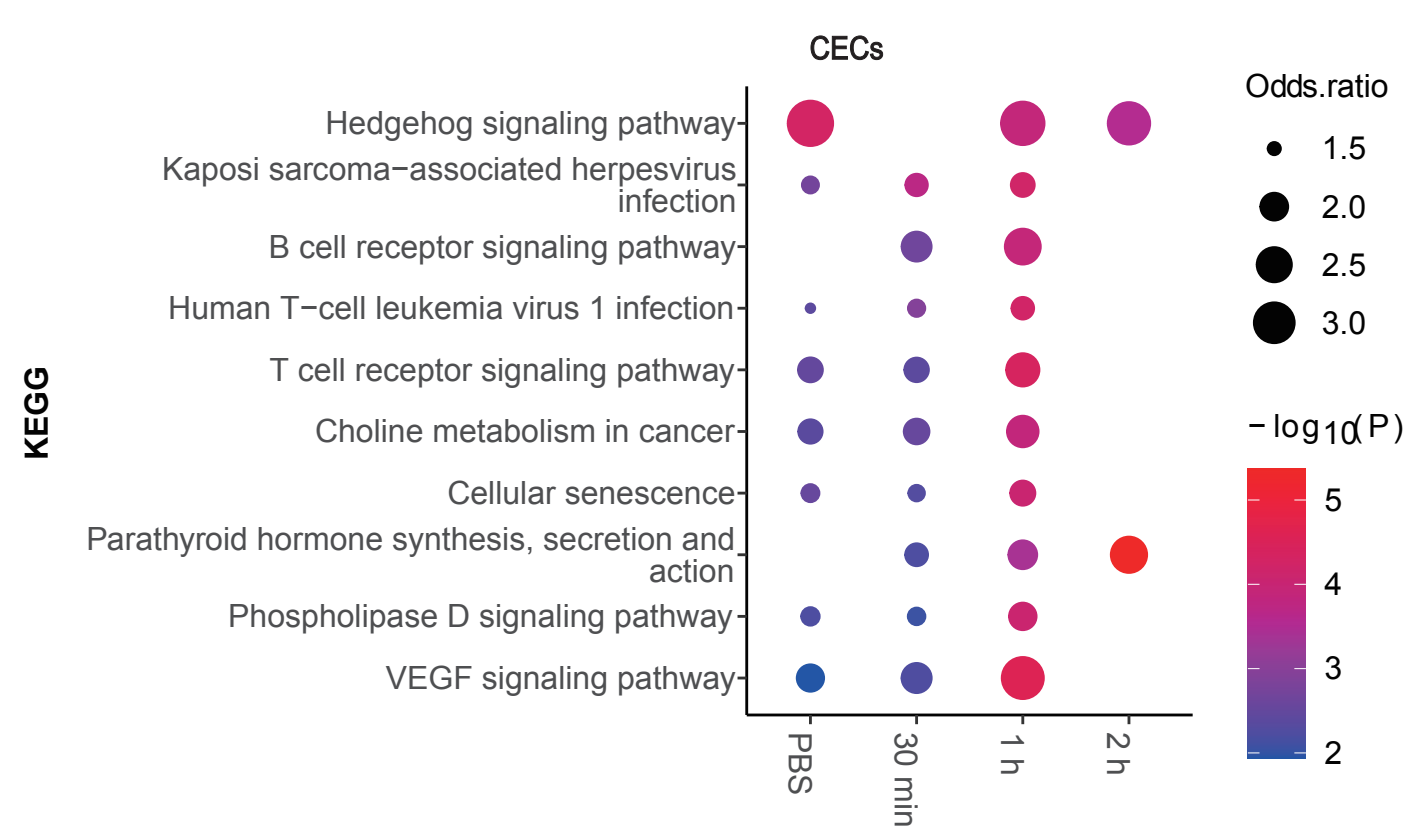**E**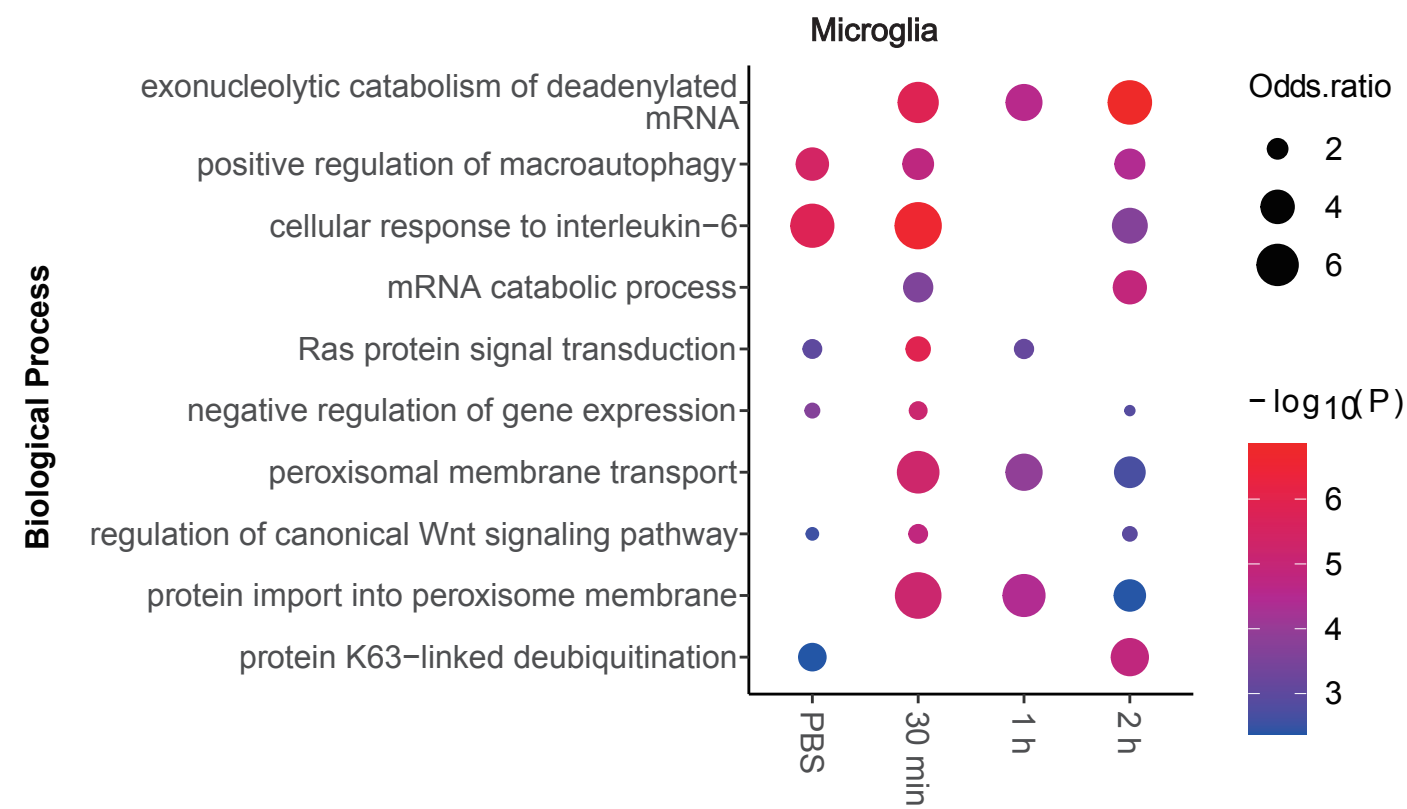**F**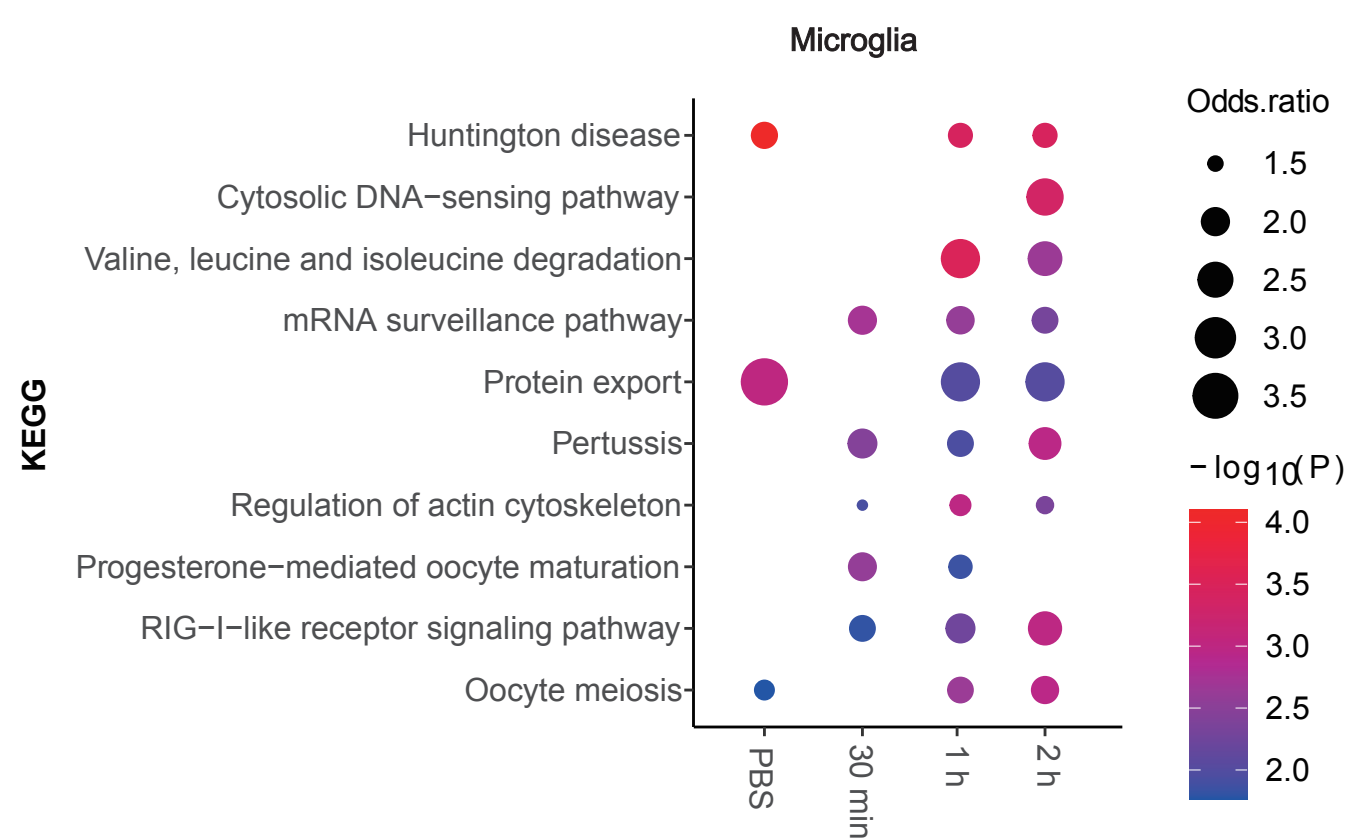

Supplement: SUPPLEMENTARY FIGURE S4 — The most differentially enriched biological processes and pathways during acute neuroinflammation. Results of GO biological processes and KEGG pathways are shown for (A,B) cerebral vessels, (C,D) CECs, and (E,F) microglia. [file Data_Sheet_4.PDF]

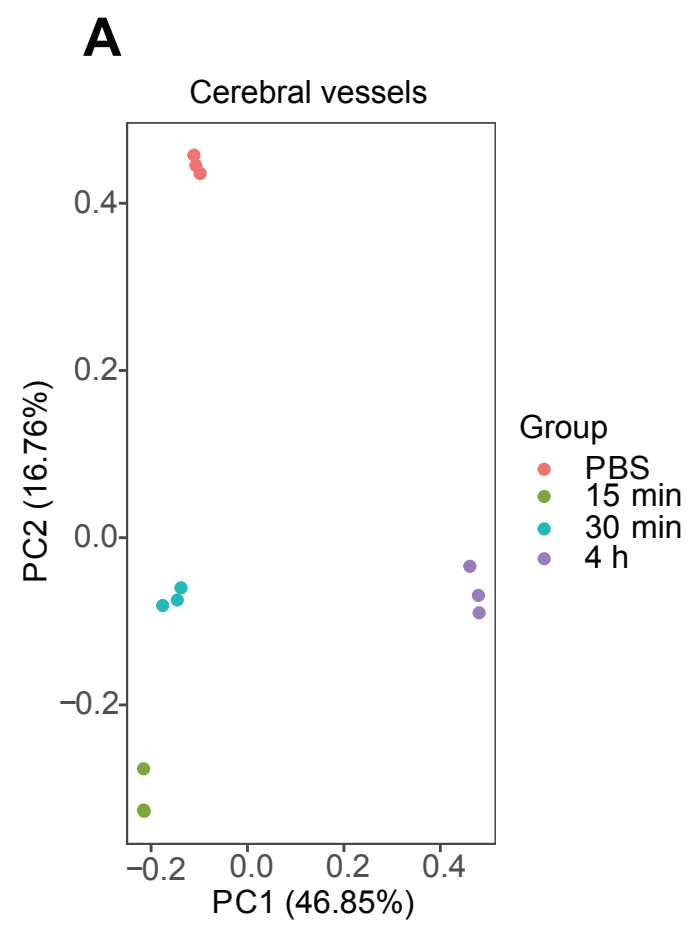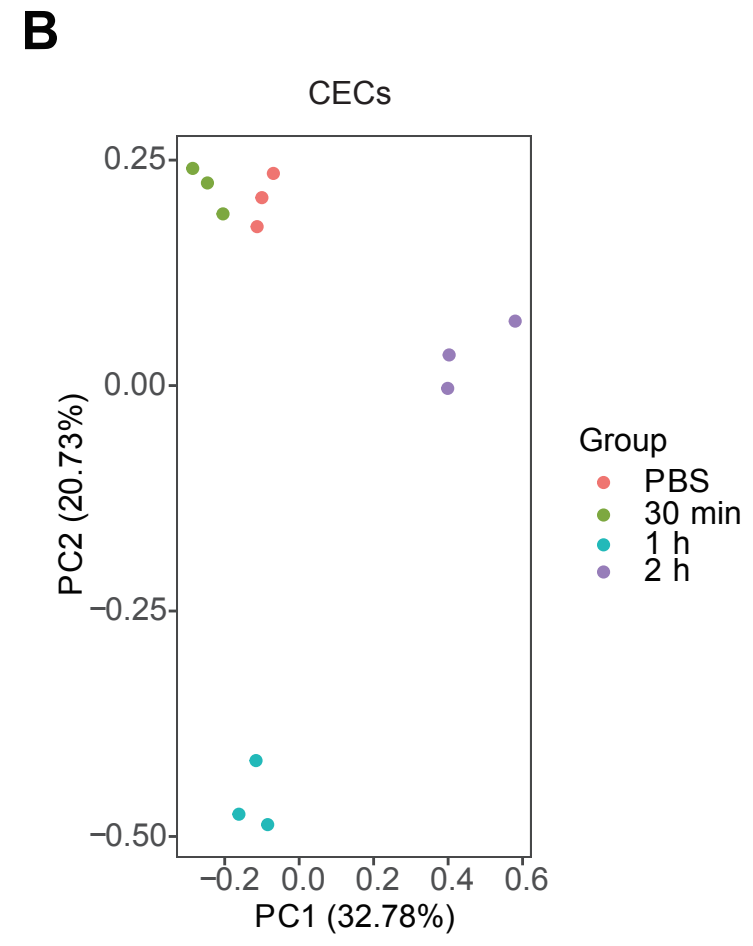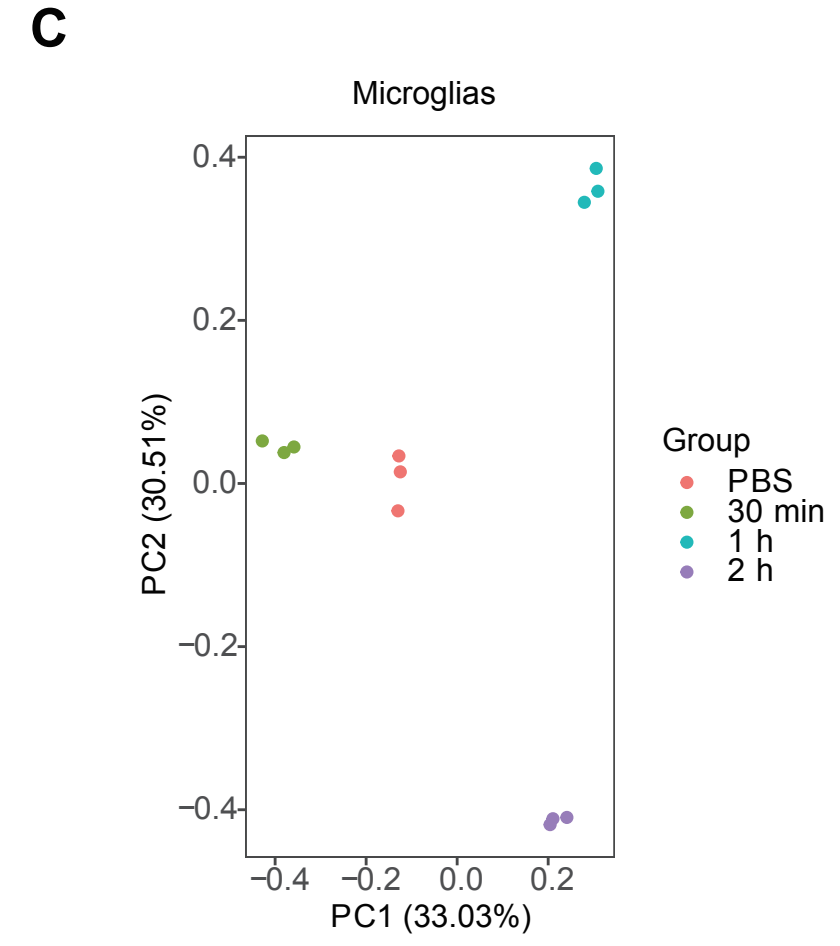

Supplement: SUPPLEMENTARY FIGURE S5 — Principle component analysis of the A-to-I RNA editing events differentially edited between different groups during acute neuroinflammation. Results are shown for (A) cerebral vessels, (B) CECs, and (C) microglia. [file Data_Sheet_5.PDF]
