# Supplementary material for: Dynamic A-to-I RNA editing during acute neuroinflammation in sepsis-associated encephalopathy
Source: Front Neurosci. 2024 Aug 2;18:1435185. doi: 10.3389/fnins.2024.1435185 (PMC11328407; doi:10.3389/fnins.2024.1435185)
Supplement: SUPPLEMENTARY FIGURE S6 — The results of overlapping RBP binding sites of Gch1:chr14:47155050 using the RBPmap. [file Data_Sheet_6.PDF]

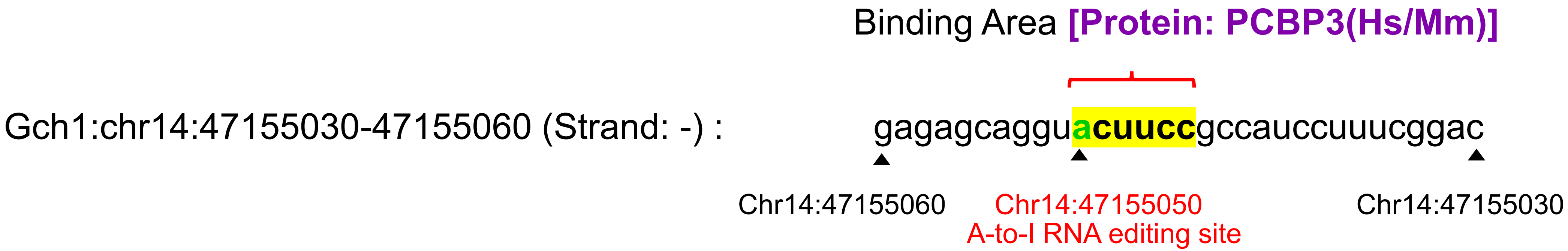
Binding Area [Protein: PCBP3(Hs/Mm)]
Gch1:chr14:47155030-47155060 (Strand: -) :
gagagcaggu**acuucc**gccauccuuucggac
Chr14:47155060
Chr14:47155050
A-to-I RNA editing site
Chr14:47155030
